# *In vitro* comparison of conventional hyperthermia and modulated electro-hyperthermia

**DOI:** 10.18632/oncotarget.11444

**Published:** 2016-08-20

**Authors:** Kai-Lin Yang, Cheng-Chung Huang, Mau-Shin Chi, Hsin-Chien Chiang, Yu-Shan Wang, Chien-Chung Hsia, Gabor Andocs, Hsin-Ell Wang, Kwan-Hwa Chi

**Affiliations:** ^1^ Department of Radiation Therapy and Oncology, Shin Kong Wu Ho-Su Memorial Hospital, Taipei, Taiwan; ^2^ Department of Biomedical Imaging and Radiological Sciences, National Yang-Ming University, Taipei, Taiwan; ^3^ School of Medicine, Fu Jen Catholic University, New Taipei, Taiwan; ^4^ Institute of Nuclear Energy Research, Taoyuan, Taiwan; ^5^ Department of Radiological Sciences, Graduate School of Medicine and Pharmaceutical Sciences, University of Toyama, Japan; ^6^ Institute of Veterinary Clinical Science, School of Veterinary Medicine, National Taiwan University, Taipei, Taiwan

**Keywords:** modulated electro-hyperthermia, conventional capacitive coupling hyperthermia, water bath, cytotoxicity, cell membrane

## Abstract

Radiofrequency-induced hyperthermia (HT) treatments for cancer include conventional capacitive coupling hyperthermia (cCHT) and modulated electro-hyperthermia (mEHT). In this study, we directly compared these methods with regard to *in vitro* cytotoxicity and mechanisms of action under isothermal conditions. Hepatoma (HepG2) cells were exposed to HT treatment (42°C for 30 min) using mEHT, cCHT or a water bath. mEHT produced a much higher apoptosis rate (43.1% ± 5.8%) than cCHT (10.0% ± 0.6%), the water bath (8.4% ± 1.7%) or a 37°C control (6.6% ± 1.1%). The apoptosis-inducing effect of mEHT at 42°C was similar to that achieved with a water bath at 46°C. mEHT also increased expression of caspase-3, 8 and 9. All three hyperthermia methods increased intracellular heat shock protein 70 (Hsp70) levels, but only mEHT greatly increased the release of Hsp70 from cells. Calreticulin and E-cadherin levels in the cell membrane also increased after mEHT treatment, but not after cCHT or water bath. These results suggest that mEHT selectively deposits energy on the cell membrane and may be a useful treatment modality that targets cancer cell membranes.

## INTRODUCTION

Hyperthermia (HT), which involves the use of different techniques to achieve a fever-like temperature (≤ 42°C) around tumors [3, 4], has been used mainly in combination with radiotherapy or chemotherapy as a cancer treatment [1, 2]. Radiofrequency (RF) treatments, in which a pair of capacitive electrodes are placed on opposite sides of the body, are the most common methods for heating deep-seated tumors. Conventional capacitive coupling hyperthermia (cCHT), delivered using devices like the Thermotron RF-8, has been widely used in oncological practice for more than 2 decades in Japan. This device produces dielectric heat with high power via rapid changes in the electric field (8 MHz) to reach a goal temperature in a specific region [5]. However, maintaining a temperature of ≤ 42°C for 30 to 60 minutes is generally not enough to cause the desired cytotoxicity. A previous study reported that, while cCHT alone resulted in complete responses in 14 out of 187 cases (7.5%), cCHT in conjunction with either radiotherapy, chemo-radiotherapy, or chemotherapy resulted in complete responses in 134 out of 270 (49.6%), 92 out of 244 (37.7%), and 54 out of 360 (15.0%) cases, respectively [6].

Modulated electro-hyperthermia (mEHT, trade name Oncothermia) is a new loco-regional electromagnetic hyperthermia method that uses a capacitive-impedance coupled 13.56 MHz RF current to selectively target malignant cells [7–9]. mEHT has been used clinically for more than 2 decades in Europe [10–12]. The energy of the RF current is selectively absorbed by the tumor tissue due to higher ionic concentrations in the tumor milieu, causing massive apoptotic cell death even below the cytotoxic temperature range [8, 9, 13, 14]. Although both mEHT and cCHT are based on similar technical principles, mEHT incorporates several technical and biomedical modifications. Due to its unique impedance-matching system based on capacitive-impedance coupling technology under low power, mEHT selectively deposits energy on malignant cell membranes [14]. This highly-selective nanoscopic heating, especially of membrane raft domains, activates different signal transduction pathways and results in programmed cell death rather than massive necrosis, which often occurs with conventional hyperthermia treatments [8, 15]. Many studies have investigated the mechanisms underlying mEHT [4, 7–9, 13–16]. A clinical case report of a stage IIIB non-small-cell lung cancer patient found that mEHT together with local radiotherapy resulted in unexpectedly long survival [17]. Phase II studies have demonstrated that mEHT is clinically beneficial and minimally toxic in patients with relapsed malignant glioma [11, 18], advanced hepatocellular carcinoma [19, 20], and advanced colorectal cancer with liver metastasis [21].

Because it is difficult to use clinical devices in preclinical studies, water baths are commonly used to study HT treatments *in vitro* and *in vivo* [22–24]. However, the only parameters that can be evaluated using a water bath are temperature and duration of treatment rather than “energy dose,” and non-thermal effects cannot be examined. Additionally, differences in the biological effects induced by various HT treatments (including water bath, cCHT, and mEHT) under isothermal conditions have not been investigated. In this report, we established standard experimental procedures for comparing these HT methods *in vitro*. We found that short-duration mEHT treatment induced stronger cytotoxic effects and increased the release of danger signals to a greater degree than other HT methods.

## RESULTS

### mEHT treatment induces apoptotic cell death

**Figure 1:**
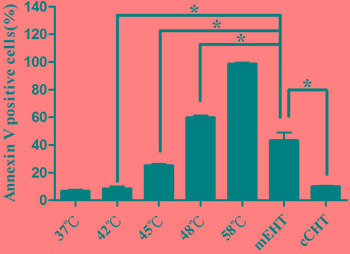
Induction of Annexin-V staining after hyperthermia treatments HepG2 cells were treated with a water bath, cCHT, or mEHT at 42°C for 30 min. Apoptosis was measured using flow cytometry after staining with FITC-conjugated Annexin V and propidium iodide. Positively-stained cells were counted using FACSCalibur. Histograms of the percentages of Annexin-V-positive cells are shown. Results from 3 independent experiments are shown; bars indicate mean ± standard deviation (SD). (*p < 0.05)

**Figure 2:**
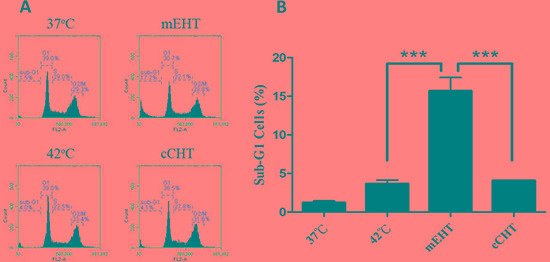
Cell cycle distribution after hyperthermia treatments HepG2 cells were treated with a water bath, cCHT, or mEHT at 42°C for 30 min. Cell cycle distribution was measured using flow cytometry after staining with propidium iodide. (**A**) Representative flow cytometric analysis plots. (**B**) Histograms of the percentages of sub-G1 cells. Results from 3 independent experiments are shown; bars indicate mean ± SD. (***p < 0.001).

Following continuous growth at 37°C, HepG2 cells were transferred to a plastic bag for treatment with mEHT, cCHT, or a water bath at 42°C for 30 min, while control cell monolayers were maintained at 37°C. Twenty-four hours after treatment, apoptosis was evaluated using FITC-conjugated Annexin V and propidium iodide reagents. A significantly higher proportion of apoptotic cells was observed after mEHT treatment (43.1% ± 5.8%) than after cCHT (10.0% ± 0.6%), 42°C water bath (8.4% ± 1.7%), or 37°C control (6.6% ± 1.1%) treatments (Figure 1). Similarly, mEHT induced a 15.67% ± 1.76% increase in the sub-G1 population, whereas cCHT and the 42°C water bath induced only slight increases in the sub-G1 population (4.1% ± 0.0% and 3.65% ± 0.49%, respectively) without altering cell cycle arrest (Figure 2). These results indicate that mEHT is more effective than conventional HT at the same temperature and duration. These results were confirmed in three additional human cancer cell lines: a breast cancer cell line (MCF7), a colon cancer cell line (WiDr), and a brain tumor cell line (U87MG). Apoptosis rates increased in all of these human cancer cell lines after mEHT compared to the water bath and cCHT treatments (Supplementary Figure S1).

Increasing the water bath temperature proportionally increased percentages of apoptotic HepG2 cells, which were 8.4% ± 1.7%, 25.1% ± 1.2%, 59.7% ± 1.5%, and 98.5% ± 1.0% for 42°C, 45°C, 48°C, and 58°C, respectively (Figure 1). Additionally, cCHT at 42°C and water bath at 42°C resulted in similar apoptosis rates, as did mEHT at 42°C and water bath at 45°C to 48°C (approximately 46°C using interpolation). A water bath at 58°C, which causes tumor ablation, served as positive control and resulted in almost complete apoptosis.

### mEHT treatment increases ROS levels in HepG2 cells

**Figure 3:**
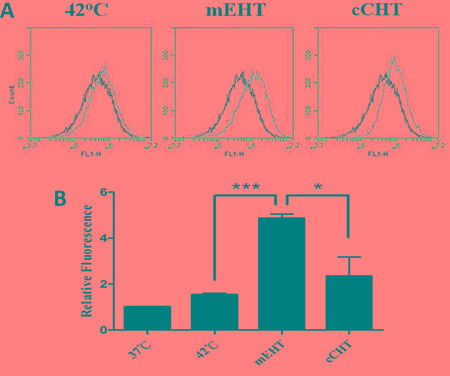
ROS levels after hyperthermia treatments HepG2 cells were treated using the different hyperthermia methods at 42°C for 30 min. 3 h after hyperthermia treatment, HepG2 cells were labeled with 5 μM dihydroethidium for 30 min and the mean fluorescence intensity of each sample was determined by flow cytometry to estimate ROS levels. (**A**) Representative flow cytometric analysis plots. (**B**) Histograms of the relative fluorescence of ROS-positive cells. Results from 3 independent experiments are shown; bars indicate mean ± SD. (*p < 0.05; ***p < 0.001).

It has been reported that HT may enhance the production of intracellular reactive oxygen species (ROS) [25], and HT-induced oxidative stress is crucial in the initiation of apoptotic cell death [26]. To investigate whether mEHT increases intracellular ROS levels in HepG2 cells, ROS levels were determined using DCFDA, an indicator of total cellular ROS. ROS production increased 3 h after mEHT (4.87 ± 0.18, Figure 3), while cCHT induced a slight, but insignificant, increase in ROS levels (2.35 ± 0.82), compared to the water bath (1.54 ± 0.06).

### mEHT treatment activates the caspase signaling pathway

**Figure 4:**
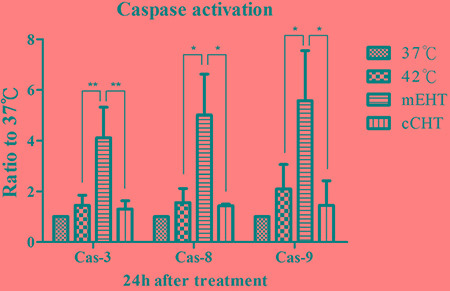
Caspase signaling after hyperthermia treatments HepG2 cells were treated using the different hyperthermia methods at 42°C for 30 min. After the indicated incubation times, cells were harvested for caspase analysis. Activated caspase 3, 8, and 9 levels were estimated in HepG2 cells using the CaspFlow™ Fluorescein Active Caspase-3, 8, 9 staining kit (BioVision) as per the manufacturer's instructions and using annexin V-FITC labeling under the same conditions described above. Results of 3 independent experiments are shown; bars indicate mean ± SD. (*p < 0.05; **p < 0.01).

Changes in caspase activation in HepG2 cells were evaluated 24 h after different HT treatments. mEHT, but not cCHT or water bath, increased expression of fluorescein-active caspase 3, 8, and 9 (Figure 4). These results suggest that mEHT induces apoptosis via a caspase-dependent pathway.

### mEHT treatment upregulates calreticulin expression

**Figure 5:**
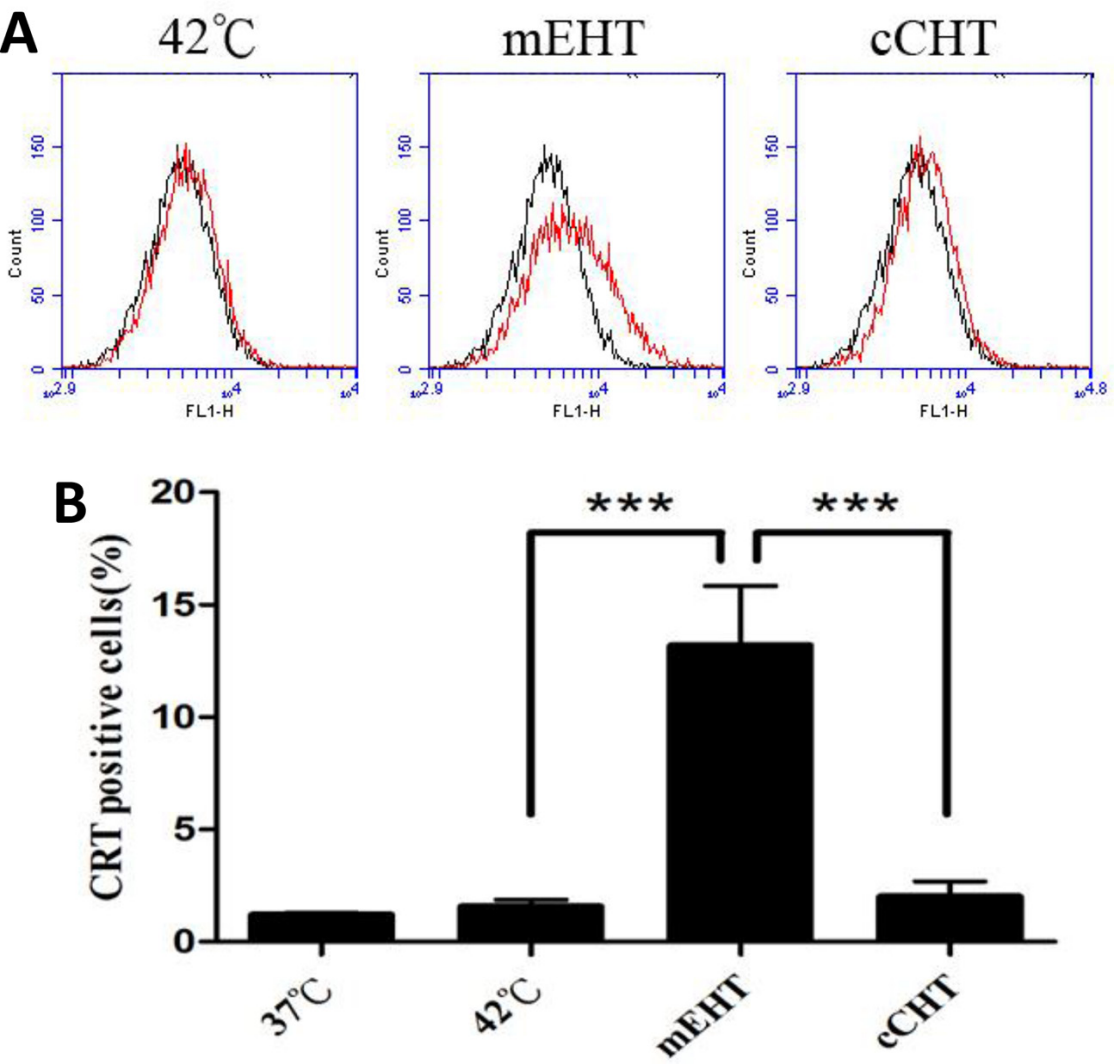
Calreticulin expression after hyperthermia treatments HepG2 cells were treated with a water bath, cCHT, or mEHT at 42°C for 30 min. After 24 h of incubation, hyperthermia-treated HepG2 cells were analyzed for calreticulin expression by flow cytometry. (**A**) Representative flow cytometric analysis plots. (**B**) Histograms of calreticulin-positive cells. Results from 3 independent experiments are shown; bars indicate mean ± SD. (***p < 0.001).

Because mEHT and heat shock treatment activate calcium channels and increase calreticulin expression [27, 28], we compared the ability of different HT treatments to induce calreticulin expression. Calreticulin expression on the cell surface increased to 13.2% ± 2.65% (Figure 5) after 30 min of mEHT at 42°C; cCHT and water bath did not increase calreticulin levels (2.03% ± 0.67% and 1.57% ± 0.31%, respectively).

### Intracellular and extracellular Hsp70 levels after HT treatments

**Figure 6:**
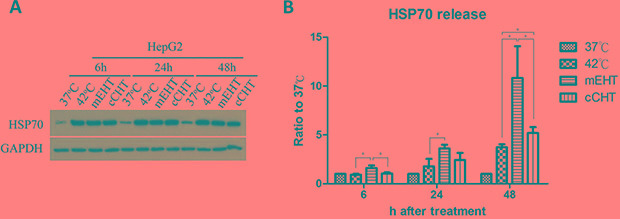
Intracellular and extracellular Hsp70 levels after hyperthermia treatments HepG2 cells were treated with a water bath, cCHT, or mEHT at 42°C for 30 min or with normal control conditions (37°C). (**A**) Intracellular Hsp70 levels in HepG2 cells (5 × 10^5^ cells) were assayed by western blot after the indicated incubation times. GAPDH was used as internal control. (**B**) Extracellular Hsp70 levels in supernatants were assayed by ELISA after the indicated incubation times. Results from 3 independent experiments are shown; bars indicate mean ± SD. (*p < 0.05).

Since increased Hsp70 expression is a hallmark of HT treatment, we examined intracellular Hsp70 levels and extracellular Hsp70 release after treatment. Whole-cell extracts were used in western blots to detect intracellular Hsp70, while released Hsp70 was extracted from cell culture supernatant and assayed by ELISA. Hsp70 protein levels were relatively low under control (37°C) conditions, but increased more than 5-fold between 6 and 48 h after all HT treatments (Figure 6A). Levels of glyceraldehyde-3-phosphate dehydrogenase (GAPDH), a housekeeping protein, were unaltered after HT, indicating that the heat shock response was specific. Most importantly, mEHT increased extracellular Hsp70 release 10.8 ± 3.23-fold 48 h after treatment compared to the 37ºC control, while cCHT and the water bath did not increase Hsp70 release (Figure 6B). Thus, although all forms of HT used in this study increased intracellular Hsp70 levels, only mEHT induced extracellular Hsp70 release.

### mEHT increases adherent protein levels

**Figure 7:**
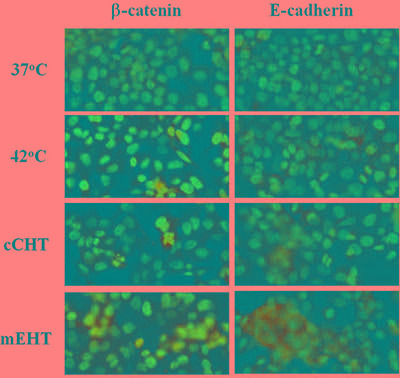
Differences in adherent protein levels after hyperthermia treatments Differences in surface β-catenin and E-cadherin levels after water bath, cCHT, or mEHT treatment at 42°C for 30 min and in normal controls (37°C). After 24 h of incubation, hyperthermia-treated HepG2 cells were fixed and stained and expression patterns were analyzed by fluorescence microscopy.

mEHT blocks tumor dissemination and inhibits the motility that results from ‘lazy’ connections within the tumor by reestablishing cellular connections [25]. Here, we investigated whether the different HT treatments restored cellular connections. As shown in Figure 7, mEHT, but not cCHT or the water bath, increased levels of adherent cell connection proteins (E-cadherin and β-catenin).

## DISCUSSION

Different HT treatments are generally thought to have similar efficacies, but direct comparisons of these treatments are lacking. In this study, we compared the biological effects of the water bath, Thermotron RF-8 (8 MHz, cCHT), and Oncotherm-LabEHY (13.56 MHz, mEHT) methods on cancer cells *in vitro*. Under isothermal conditions (42°C for 30 min), we found that mEHT increased apoptosis rates more than the other HT methods. Additionally, mEHT alone increased caspase-3, 8, and 9 activation, calreticulin expression, extracellular Hsp70 release, and cell-cell adhesion molecule levels. These results indicate that mEHT triggers anti-tumor responses on the cell membrane.

At least 2 kinds of radiofrequency hyperthermia (RF-HT) machines, cCHT and mEHT, are currently available. The cCHT method requires a large amount of power to heat a large region, and does not allow for selective heating of the tumor site. In contrast, the mEHT method takes advantage of elevated conductivity, permittivity, and current density in tumors to specifically direct RF current flow through the tumor site, and thus requires less power. An RF current with well-matched impedance self-focuses the 13.56 MHz stimulation deep within the tumor site. These engineering and physical properties increase the electrical and thermal effects of the treatment. Many studies have characterized the effects of hyperthermia treatments *in vivo* [8, 15, 28–31]. Water baths exert thermal effects alone, while RF-HT treatments also have non-thermal tumoricidal effects. Here, we found that cCHT at 42°C increased apoptotic rates slightly more than a water bath at 42°C, while mEHT at 42°C increased apoptotic rates substantially more than a water bath between 45°C and 48°C (approximately 46°C). This result confirmed that RF-HT has non-thermal effects, and that these effects were stronger with mEHT than with cCHT. The mechanisms underlying these non-thermal effects of mEHT require further investigation.

mEHT induced apoptotic cell death in HT29 colorectal cancer xenografts in a BALB/c (nu/nu) mouse model via activation of apoptosis-inducing factor (AIF) [32]. However, cytoplasmic release of cytochrome c did not result in caspase-3 activation in that study [32]. In contrast, we found that both extrinsic (caspase-8) and intrinsic (caspase-9) caspase-dependent pathways were activated *in vitro* 24 h after mEHT. Differences in the experimental models (*in vitro* versus *in vivo*) and cell lines used might account for these conflicting results. *Invivo* models are much more complex than *in vitro* systems due to the presence of normal tissues and immune cells around the tumor site, a relatively intact extracellular matrix, and cell-cell adhesion mechanisms. In addition, interplay between immune cells and extrinsic apoptotic pathway regulation can complicate the interpretation of results obtained using *in vivo* models [33]. Moreover, cross-talk between extrinsic and intrinsic apoptosis pathways increases the complexity of such studies [34, 35]. Here, ROS-induced mitochondrial damage did not increase 1 h after HT, regardless of the method used (data not shown). However, ROS production increased 4.8-fold 3 h after mEHT and 2.4-fold after cCHT compared to the water bath treatment. These results confirm that RF treatment may exert an additional electromagnetic effect on lipid-rich organelles [36], and the resultant oxidative stress likely induces apoptosis [26].

The ability of mEHT to induce apoptosis in a high percentage of tumor cells and to increase the release of Hsp70 is thought to be crucial for tumor-specific immune response. In our previous study using a CT-26 murine colon cancer model, co-injection of rHsp70 and dendritic cells (DCs) into the irradiated tumor site triggered a more potent anti-tumor immune response than DCs alone by converting radiation-induced local apoptosis into a systemic antitumor immune response [37]. In the present study, mEHT increased the release of Hsp70 from heated tumors into the extracellular space to a greater degree than the other HT methods. We previously found that Hsp70 release serves as a danger signal, increasing immunological responsiveness and the infiltration of eosinophils in the tumor microenvironment, in the *in vivo* CT-26 murine colon cancer model [37]. Additionally, many cancer cells exhibit surface calreticulin expression, which may promote phagocytosis by macrophages [38]. Here, mEHT increased calreticulin expression on the cell surface, likely promoting anti-cancer immune response.

Notably, overexpression of adhesion molecules (e.g. β-catenin and E-cadherin) was only observed after mEHT treatment, reflecting another difference between mEHT and other HT methods. The mechanisms underlying adhesion molecule overexpression after mEHT have not yet been identified. However, increased calreticulin expression, Hsp70 release, and overexpression of adhesion molecules all indicate that energy deposition at the cell membrane may be crucial for the early actions of mEHT. Therefore, the main advantage of mEHT may be its ability to selective heat tumor cell membranes as well as the cytosol and interstitial space. Moreover, a temperature measurement of 42°C in the interstitial space may not accurately reflect gains in energy deposited on the cell membrane in other HT methods.

In conclusion, our results indicate that mEHT induces apoptosis more efficiently than cCHT or a water bath under isothermal conditions. Additionally, mEHT likely deposits energy specifically on the cell membrane. The increased immunostimulatory effects of mEHT may be due to increases in calreticulin levels and Hsp release. Effects on the cell membrane should be considered during hyperthermia treatment, and mEHT may be a valuable new treatment modality due to its ability to target cancer cell membranes.

## MATERIALS AND METHODS

### Cell culture

The HepG2 hepatoma cell line was maintained in Dulbecco's Modified Eagle Medium (DMEM; Invitrogen, Verviers, Belgium) containing 10% heat-inactivated fetal bovine serum (FBS), 2 mM L-glutamine, 100 units/mL penicillin, and 100 μg/mL streptomycin (Sigma, St. Louis, MO). Additional human cancer cell lines, the MCF7 breast cancer cell line, the WiDr colon cancer cell line, and the U87MG brain tumor cell line, were used to confirm the effects of hyperthermia treatments.

### mEHT treatment

**Figure 8:**
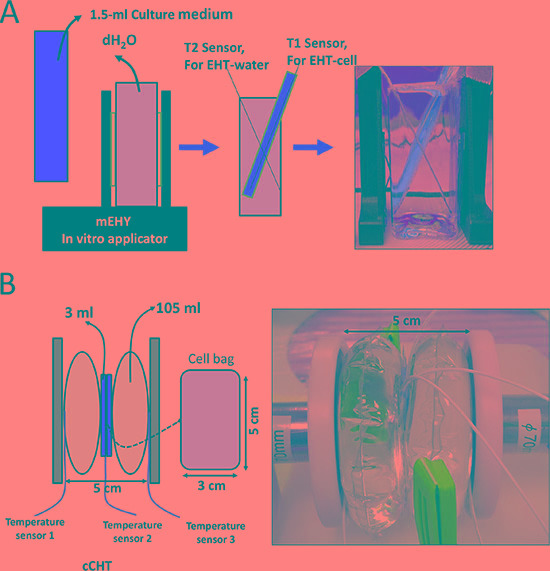
*In* *vitro* HT exposure experimental setups (**A**) Oncotherm LabEHY, (**B**) Thermotron RF-8. HepG2 cells were treated at 42°C for 30 min.

Electromagnetic heating was generated using a capacitively-coupled, amplitude-modulated, 13.56-MHz radiofrequency (LabEHY, Oncotherm Ltd, Troisdorf, Germany). An *in vitro* heating model was established in an electrode chamber (LabEHY *in vitro* applicator). The chamber contained a cell bag (1 × 10^6^ cells) heated to 42°C for 30 min with an average power of 10 to 12 W. Temperature was maintained at approximately 42°C on the treated side as measured with optical sensors (Luxtron FOT Lab Kit, LumaSense Technologies, Inc., California, USA). The *in vitro* model setup is schematically illustrated in Figure 8A. The power pattern was checked each time to verify the accuracy and similarity of the experiments. Power patterns for three separate runs are shown in Supplementary Figure S2. In a previous study, Andocs *et* *al*. demonstrated the precision of the mEHT method [39]. For water bath treatment, 1 × 10^6^ cells were placed in a tube with culture medium and incubated at 42°C for 30 min.

mEHT treatment conditions were based on previous publications [8, 15, 16, 40]. Cha *et* *al*. treated cells for 60 minutes [26], but we previously found that 30-minute treatment times were sufficient to produce biological responses [16, 27–29, 41]. We therefore used 30-minute treatment times here. Although classic hyperthermia treatments at 42°C were not sufficient to produce significant cell death in our previous study [42], mEHT at this temperature induces significant biological responses due to its unique mechanism of action; we therefore conducted mEHT treatments at 42ºC.

### cCHT treatment

cCHT was conducted using an 8-MHz RF capacitive heating device (Thermotron RF-8; Yamamoto Vinita Co., Osaka, Japan). In this system, the RF generator contains a self-excited oscillation circuit set at 8 MHz and 1.5 kW maximum output power. RF energy is transmitted from a generator via 2 coaxial cables to 2 disc electrodes. RF was applied through a pair of electrodes placed on opposite sides of the cell bag and power was distributed regionally via rapid changes in electric fields produced between the parallel-opposed electrodes. The cell bag (1 × 10^6^ cells) was heated to 42°C for 30 min at an average power of 16 to 19 W (Forward power-Reflected power). Temperature was maintained at approximately 42°C on the treated side as measured with optical temperature sensors installed on the device. The *in vitro* model set-up is schematically illustrated in Figure 8B. The power pattern was checked each time to verify the accuracy and similarity of the experiments.

### Apoptosis assay

HepG2 cells (5 × 10^5^ cells) treated using the water bath, cCHT, or mEHT were seeded on 6-well plates, cultured for 24 h, trypsinized, and washed twice with phosphate-buffered saline (PBS). Apoptosis was confirmed using an Annexin-V Apoptosis Kit (BD Pharmingen) according to the manufacturer's instructions. Briefly, tumor cells were washed 3 times with PBS and stained with Annexin V and propidium iodide, incubated in the dark on ice for 10 min, and analyzed by flow cytometry. The percentage of positive cells was determined using a FACSCalibur cytometer and Cell Quest Pro software (Becton Dickinson, Mountain View, CA). mEHT-induced apoptosis rates were also confirmed using an Annexin-V assay in all subsequent experiments investigating effects other than apoptosis.

### Sub-G1 cell cycle analysis

After incubation for 24 h, HepG2 cells (1 × 10^6^ cells) with or without HT treatment (water bath, cCHT, or mEHT) were trypsinized and washed twice with PBS. Cell pellets were suspended in 1 mL 70% ethanol for 30 min at –20°C. Cells were centrifuged, resuspended in 1 mL of propidium iodide staining solution (0.04 mg/mL propidium iodide, 100 μg/mL DNase-free RNase A), and incubated at 37°C for 20 min. Flow cytometric analysis was performed using a FACSCalibur cytometer.

### Assay for caspase-like activity

Caspase-like activity was evaluated using a CaspGLOW™ Fluorescein Active Caspase-3, 8, 9 Staining Kit (BioVision, Milpitas, CA, USA) according to the manufacturer's instructions. The kit detects active caspases in living cells using fluorescein isothiocyanate (FITC)-labeled-Asp-Glu-Val-Asp-fluoromethylketone (DEVD-FMK), which permeates cells and binds irreversibly to active caspase-3, 8, and 9. Briefly, HepG2 cells with or without HT treatment (water bath, cCHT, or mEHT) were incubated for the indicated times, and 3 × 10^5^ cells were then incubated for 1 h with FITC-DEVD-FMK at 37°C. Subsequently, cells were washed twice with washing buffer and fluorescence intensity was measured using a FACSCalibur cytometer.

### Measurement of total cellular reactive oxygen species (ROS) levels

Total cellular ROS levels were examined using 2′,7′–dichlorofluorescin diacetate (DCFDA) according to the manufacturer's instructions. Briefly, HepG2 cells (1×10^6^ cells) treated for 3 h with or without HT were washed twice with PBS, re-suspended in PBS supplemented with 0.25 μM H_2_DCFDA, and incubated in the dark for 30 min at 37°C. The cells were analyzed by FACSCalibur cytometry.

### Western blot analysis

For intracellular protein analysis, water bath-, cCHT-, and mEHT-treated HepG2 cells (5 × 10^5^ cells) were incubated for the indicated times and dissolved in Radioimmunoprecipitation assay (RIPA) buffer (Sigma) with ethylenediaminetetraacetic acid-free Protease Inhibitor Cocktail Tablets and Phosphatase Inhibitor Cocktail Tablets (Roche). Total protein concentration was measured in lysates using the bicinchoninic acid (Pierce) protein concentration assay. Total protein (20 μg) was electrophoresed on 10% polyacrylamide gels, transferred onto Immobilon-P polyvinylidene fluoride membranes (Millipore, Bedford, MA), and blocked with Tris-buffered saline (TBS)-Tween 20 and 5% non-fat milk for 1 h at room temperature. Filters were probed with anti-Hsp70 (Santa Cruz Biotechnology, Santa Cruz, CA) or anti-GAPDH (Sigma) antibodies at 4°C overnight in TBS-0.05% Tween 20 containing 5% non-fat milk followed by 1 h incubation at room temperature with horseradish peroxidase-conjugated secondary antibodies (Jackson ImmunoResearch Laboratories, West Grove, PA) in the same buffer. Blots were developed using a chemiluminescent detection system (ECL; GE Life Science, Buckinghamshire, UK).

### Hsp70 release assay

Water bath-, cCHT-, and mEHT-treated HepG2 cells (5 × 10^5^ cells) were seeded on 6-well plates in DMEM (2 ml) containing 10% FBS and cultured for the indicated times at 37°C under 5% CO_2_. Culture supernatant was harvested and Hsp70 was measured using an enzyme-linked immunosorbent assay (ELISA) (Enzo Life Sciences, Farmingdale, USA). A Multiskan Plus device (Thermo Scientific, Hudson, NH, USA) was used to measure absorbance at 450 nm.

### Evaluation of calreticulin (CRT) expression

CRT expression on the cell surface was evaluated using indirect immunofluorescence analysis, in which 1 × 10^5^ cells were washed twice with fluorescence-activated cell sorter (FACS) buffer (2% FBS and 0.02% sodium azide in PBS, pH 7.4) and incubated with isotype control or anti-CRT mouse monoclonal antibody (Abcam, ab22683). Cells were then washed and stained with FITC-conjugated goat anti-mouse IgG (BD Pharmingen, San Diego, CA, USA) for 30 min. Finally, all cells were washed and suspended in FACS buffer containing 5 μg/ mL propidium iodide. The surface immunofluorescence of 1 × 10^4^ viable cells was measured by FACSCalibur cytometry.

### Immunofluorescence staining

Cells were co-cultured as described in the previous section, plated on glass slides, and immediately fixed with 3.7% paraformaldehyde for 15 min; this and all subsequent steps were performed at room temperature. The cells were washed once with PBS, blocked with blocking buffer (PBS + 3% BSA) for 30 min, and permeabilized by incubation with 0.1% Triton X-100 (Sigma) in PBS for 15 min. For beta-catenin staining, cells were washed once with PBS, incubated for 16 h with goat anti-human beta-catenin (R&D systems) at 4°C, and then stained with FITC-conjugated donkey anti-goat IgG (H&L) (Abcam) for 60 min at room temperature. For E-Cadherin staining, cells were directly stained with Alexa Fluor 488-conjugated anti-human E-cadherin (24E10) rabbit monoclonal antibody (Cell signaling) for 16 h at 4°C, followed by 5 washes in PBS containing 0.05% Tween 20. The slides were then mounted in aqueous mounting solution with coverglasses (Fisher Scientific) before confocal fluorescent microscopy. Images were acquired using an Olympus FV1000 microscope (Melville, NY, USA) equipped with a digital camera.

### Statistical analysis

All results were compared using unpaired *t*-tests (2-tailed) or one-way Analyses of Variance; *p* values < 0.05 indicated statistically significant differences.

## SUPPLEMENTARY MATERIALS FIGURES AND TABLES


