# Supplementary material for: In vitro comparison of conventional hyperthermia and modulated electro-hyperthermia
Source: Oncotarget. 2016 Aug 20;7(51):84082–92. doi: 10.18632/oncotarget.11444 (PMC5356646; doi:10.18632/oncotarget.11444)
Supplement: Supplementary file 1 [file oncotarget-07-84082-s001.pdf]

In vitro comparison of conventional hyperthermia and modulated electro-hyperthermia

Supplementary Materials

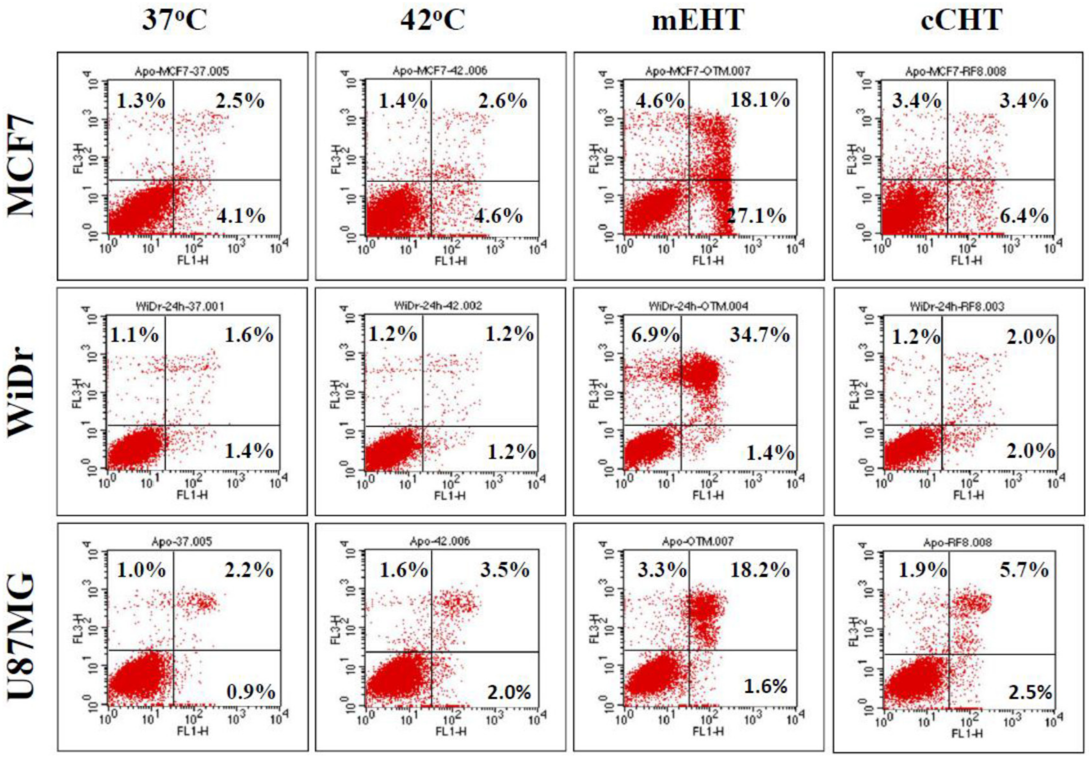

Supplementary Figure S1: Induction of Annexin-V-positive cells after hyperthermia treatment. MCF7, WiDr and U87MG cells were treated with water bath control, cCHT, or mEHT at 42°C for 30 min. Apoptosis was measured using flow cytometry after staining with FITC-conjugated Annexin V and propidium iodide. Positively stained cells were counted using FACSCalibur.

Repeat 1

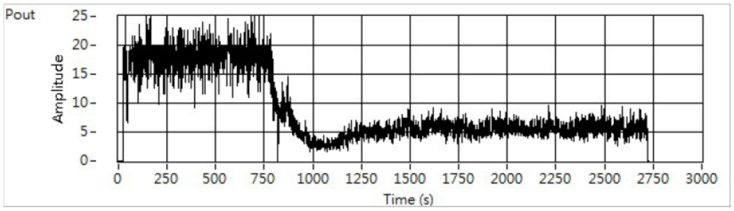

Total power:
24376.4

Repeat 2

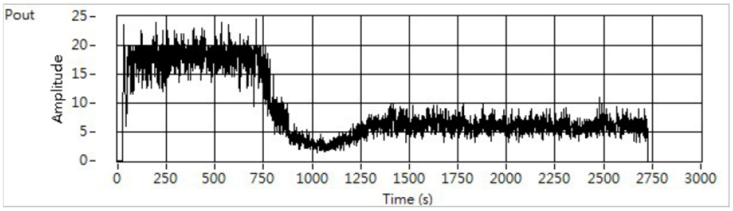

Total power:
24455.3

Repeat 3

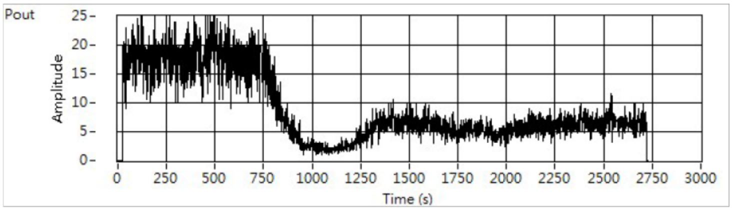

Total power:
24261.3

Supplementary Figure S2: Power pattern plots of modulated electro-hyperthermia. Power pattern plots were recorded from three independent experiments using modulated electro-hyperthermia. The total power used in this treatment was calculated and showed in right side of each plot.
